# Numbers needed to detect a significant change in myocardial strain and left ventricular twist measured by complementary spatial modulation of magnetization (CSPAMM)

**DOI:** 10.1186/1532-429X-14-S1-P259

**Published:** 2012-02-01

**Authors:** Peter Swoboda, Abdulghani M Larghat, Timothy Fairbairn, Manish Motwani, John P Greenwood, Sven Plein

**Affiliations:** 1 Department of Cardiology, Leeds General Infirmary, Leeds, UK; 2 Multidisciplinary Cardiovascular Research Centre & Leeds Institute of Genetics, Health and Therapeutics, University of Leeds, Leeds, UK

## Summary

We have calculated the inter-study reproducibility of myocardial strain and twist measured by CSPAMM and calculated the number of subjects required to detect a significant change in these parameters.

## Background

The inter-study reproducibility of parameters measured by CSPAMM such as circumferential strain, radial strain and left ventricular (LV) twist have not been described in detail and it is not known how many subjects are required to detect a significant change.

## Methods

12 healthy volunteers (6 male, mean age 33±15 years) underwent CMR studies on two separate occasions (mean interval of 8±3 days) using an identical CSPAMM pulse sequence with images acquired in 3 short axis slices at the apex, mid-ventricle and base. Data were analysed using harmonic phase analysis (Tag Track, Gyrotools, Zurich, CH). Circumferential Lagrangian strain, radial Lagrangian strain and rotation were calculated for the three short axis slices. Left ventricular (LV) twist was calculated by subtracting the basal rotation from the apical rotation. The mean difference between paired measurements, the standard deviation (SD) of the differences and the coefficient of variability (CoV) were calculated.

The sample sizes required by CMR to show a clinical change with a power of 90% and an α error of 0.05 were calculated using the formula n = f (α, P) x σ 2 x 2/ δ 2 , where n is the sample size, α the significance level, P the study power required and f the value of the factor for different values of α and P (f = 10.5 for α=0.05 and p = 0.90), with σ the inter-study standard deviation of differences between visits and δ the desired difference to be detected.

## Results

Inter-study reproducibility of circumferential strain (CoV 3.7% to 5.5%) and LV twist (CoV of 9.6%) were good (Table 1). Radial strain showed poorer inter-study reproducibility (CoV of 13.8 - 23.4%).

**Table 1:** Inter-study reproducibility as the percentage difference between the two scans and the equivalent Coefficient of Variation(%)

		Endocardium			Midline			Epicardium		
		
		Mean diff %	SD	CoV (%)	Mean diff %	SD	CoV (%)	Mean diff %	SD	CoV (%)
Circumferential strain	Apex	7.27	5.74	5.45	5.75	3.41	4.23	5.02	4.29	3.71
	Mid LV	7.41	4.02	5.50	5.32	2.64	3.89	6.09	4.15	4.50
	Base	6.44	4.21	4.77	7.14	4.43	5.31	6.50	4.56	4.83

Radial Strain	Apex				17.17	15.17	14.33			
	Mid LV				17.10	11.53	13.78			
	Base				24.98	23.40	23.40			

LV twist		13.47	10.71	10.68	12.51	9.58	9.80	15.20	11.17	12.15

Sample size calculations suggested that 20 or fewer subjects are needed to detect a 10% change in circumferential strain with a power of 90% and an α error of 0.05. For LV twist, 66 subjects would be required (Table 2).

**Table 2:** Mean values of circumferential strain, radial strain and LV twist from 12 healthy volunteers and the sample size required to detect a 10% change in each absolute value.

		Endocardium		Midline		Epicardium	
		
		Mean	Sample Size	Mean	Sample Size	Mean	Sample Size
Circumferential strain	Apex	-0.38	20	-0.29	9	-0.21	10
	Mid LV	-0.33	15	-0.24	8	-0.19	11
	Base	-0.29	14	-0.23	16	-0.18	14

Radial Strain	Apex			0.22	153		
	Mid LV			0.21	172		
	Base			0.21	436		

LV twist (degrees)		12.25	72	10.44	66	8.60	112

## Conclusions

CSPAMM tagged CMR with HARP analysis provides reproducible measurements of circumferential strain and twist with small numbers required to detect differences in serial studies. The method may provide powerful surrogate data for clinical studies.

## Funding

This research has partly been supported by a grant from the British Heart Foundation. This funding body was not involved in the study design, data collection, data analysis or writing of the manuscript.

